# Complex coronary artery fistula in a young woman presenting with chest pain

**DOI:** 10.1093/ehjcr/ytag449

**Published:** 2026-06-22

**Authors:** Tanesh Ayyalu, Zachary R Spahr, Caleb Wutawunshe, Amar Shah

**Affiliations:** Department of Cardiology, Saint Vincent Hospital, Worchester, MA 01608, USA; Department of Medicine, MedStar Georgetown University Hospital, 3800 Reservoir Rd NW, Washington, DC 20007, USA; Department of Cardiology, Lenox Hill Hospital, New York, NY 10075, USA; Department of Cardiology, Lenox Hill Hospital, New York, NY 10075, USA

## Summary

A 40-year-old woman presented with intermittent chest pain and was found on coronary computed tomography angiography to have a fistulous communication between the left anterior descending artery, left internal mammary artery, and left pulmonary artery. Invasive coronary angiography failed to re-demonstrate the anomaly, and the patient was discharged for outpatient follow-up and further dedicated shunt evaluation. This represents the first reported case of a congenital left anterior descending (LAD)– left internal mammary artery (LIMA)– left pulmonary artery (LPA) fistula, highlighting the diagnostic value of coronary computed tomography angiography (CCTA) when invasive angiography is inconclusive and the lack of standardized management guidelines for such anomalies.

**Figure 1:**
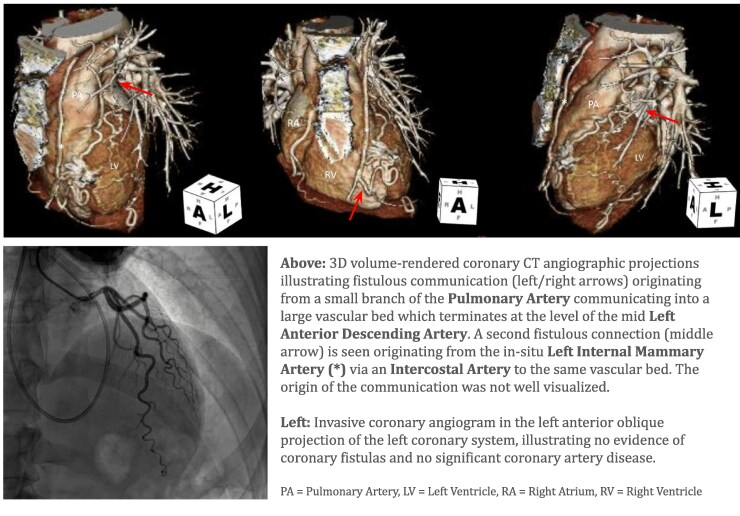
Above: 3D volume-rendered coronary CT angiographic projections illustrating fistulous communication (left/right arrows) originating from a small branch of the pulmonary artery converging into a large vascular bed which terminates at the level of the mid left anterior descending artery. A second fistulous connection (middle arrow) is seen originating from the in-situ left internal mammary artery (*) via an Intercostal Artery to the same vascular bed. The origin of the communication was not well visualized. Left: Invasive coronary angiogram in the left anterior oblique projection of the left coronary system, illustrating no evidence of coronary fistulas and no significant coronary artery disease. PA, pulmonary artery; LV, left ventricle; RA, right atrium; RV, right ventricle.

A 40-year-old female with hyperlipidaemia presented with intermittent chest pain radiating to her left arm. Her electrocardiogram revealed normal sinus rhythm with non-specific T wave changes. She had no cardiac enzyme elevation and was evaluated with CCTA due to her intermediate pre-test probability. This demonstrated no calcified or non-calcified plaque, but showed a large vascular bed in the area of the mid LAD artery (*Figure 1*), communicating with dual moderate-sized (1–2 × normal luminal size) fistulas to the LIMA via an intercostal branch, and to the LPA. Despite the fistula size there was no chamber enlargement or evidence of ischaemic changes. The patient underwent a left heart catheterization (*Figure 1*), which was unable to re-demonstrate the fistulas. Echocardiogram showed a preserved left ventricular ejection fraction. With no angiographic or biochemical evidence of coronary obstruction closure was deferred, she was discharged for outpatient follow-up with dedicated fistula shunt studies. A key limitation of this report is the absence of longitudinal follow-up, as the patient was ultimately lost to follow-up, precluding further assessment of fistula haemodynamics, progression, and clinical outcomes.

Coronary artery fistulas are rare anomalies affecting 0.1% to 0.2% of the population and are usually detected incidentally, with communications between the LIMA and LPA documented in only thirty cases.^1,2^ These can be congenital or acquired secondary to trauma, neoplastic invasion, or after coronary artery bypass grafting.^2^ This is the first documented case of a congenital fistula between the LAD, LIMA, and LPA.

Small coronary artery fistulas may be asymptomatic, but larger fistulas can lead to myocardial ischaemia (MI), arrhythmias, endarteritis, chamber enlargement, or ventricular dysfunction if untreated. There are currently no consensus guidelines for management. Transcutaneous and surgical closure methods can be complicated by post-procedural MI, vessel dissection, or haemorrhage.

In complex coronary fistulas, visualization on invasive angiography (IC) may be limited due to overlapping vascular structures, suboptimal opacification from high-flow states, or low-pressure systems. As demonstrated in this case, CCTA offers superior anatomical delineation and may identify fistulous connections not appreciated on IC. Despite advances in imaging, management of rare congenital coronary fistulas remains poorly standardized, with decisions often individualized based on anatomy, symptoms, and haemodynamic significance. Current consensus suggests repeat CCTA within five years of closure to assess vessel patency.^1^

## Data Availability

The available data is present within the body of the manuscript.

## Notes

**Consent:** Consent obtained in accordance with COPE/ICMJE recommendations and as described in EHJCR policy statements.

## References

[1] Al-Hijji M, El Sabbagh A, El Hajj S, AlKhouli M, El Sabawi B, Cabalka A, et al Coronary artery fistulas. JACC Cardiovasc Interv 2021;14:1393–1406. 34238550 10.1016/j.jcin.2021.02.044

[2] Barot TC, LaPietra A, Santana O, Beohar N, Lamelas J. Multiple left internal mammary artery-to-pulmonary artery fistulae 15 years after coronary artery bypass grafting. Tex Heart Inst J 2014;41:94–96. 24512413 10.14503/THIJ-12-3132 PMC3967476

